# Phenotypic antimicrobial resistance structure among Enterobacterales circulating at the turkey–environment interface in a small-scale poultry production system

**DOI:** 10.3389/fvets.2026.1935777

**Published:** 2026-10-15

**Authors:** Alex Yauyo, Alejandra Castro, Brenda Ayzanoa, Marcelo Cardenas, Guillermo Salvatierra

**Affiliations:** 1 Faculty of Health Sciences, Universidad Peruana de Ciencias Aplicadas, Lima, Peru; 2 One Health Research Laboratory, Faculty of Health Sciences, Universidad Privada del Norte, Lima, Peru

**Keywords:** antimicrobial resistance, carbapenem resistance, enterobacterales, multidrug resistance, one health, phenotypic clustering, poultry production systems

## Abstract

**Introduction:**

Antimicrobial resistance is a major One Health challenge affecting human, animal, and environmental health. Poultry production systems may contribute to the maintenance and dissemination of multidrug-resistant (MDR) Enterobacterales through interactions among animals and their environment. Conventional analytical approaches may have limited capacity to characterize the complexity of phenotypic resistance profiles. This study aimed to characterize phenotypic antimicrobial resistance patterns and explore resistance clustering among Enterobacterales isolated from turkeys and their production environment in Lima, Peru.

**Methods:**

A descriptive cross-sectional study was conducted using Enterobacterales recovered from cloacal swabs and environmental samples collected in a small-scale poultry production system. Antimicrobial susceptibility testing was performed using the disk diffusion method; intermediate results were classified as susceptible for analytical purposes. Multiple correspondence analysis (MCA) combined with k-means clustering was applied to explore the multivariate structure of phenotypic resistance profiles.

**Results:**

A total of 50 Enterobacterales isolates were recovered from cloacal swabs (*n* = 38; 76.0%) and environmental samples (*n* = 12; 24.0%). Predominant species included *Escherichia coli* (*n* = 39; 78.0%), followed by *Klebsiella* spp. (*n* = 7; 14.0%) and *Citrobacter* spp. (*n* = 4; 8.0%). All isolates were classified as MDR. The most frequent resistance profile included kanamycin, tetracycline, neomycin, cefuroxime, and amoxicillin–clavulanic acid. One *E. coli* isolate exhibited resistance to 18 antimicrobials, while a *Citrobacter* spp. isolate recovered from the environment showed resistance to 17 antimicrobials, including imipenem. MCA revealed a structured distribution of resistance phenotypes, with the first two dimensions explaining 45.3% of the total inertia and identifying three clusters with distinct resistance spectra. One cluster contained all carbapenem-resistant isolates and exhibited the broadest resistance profile.

**Discussion:**

These findings highlight the circulation of heterogeneous MDR populations and support surveillance strategies in poultry production environments.

## Introduction

Antimicrobial resistance represents one of the most pressing global health challenges, with substantial implications for clinical outcomes, healthcare sustainability, food security, and livestock production (1). The inappropriate and prolonged use of antimicrobials in animal production systems has contributed to the emergence and dissemination of multidrug-resistant Enterobacterales (2, 3), a group of bacteria capable of causing severe infections in both humans and animals (4, 5). A major factor underlying their epidemiological relevance is their ability to acquire and disseminate resistance determinants through horizontal gene transfer, particularly via plasmid-mediated mechanisms, facilitating the persistence of multidrug-resistant phenotypes within the human-animal-environment continuum (2, 6, 7).

*Escherichia coli* and *Klebsiella* spp. are among the most frequently reported Enterobacterales associated with antimicrobial resistance given to their ability to harbor intrinsic and acquired mechanisms affecting multiple antimicrobial classes. These bacteria are commonly implicated in gastrointestinal and opportunistic infections across diverse animal hosts (8, 9). The increasing detection of resistance determinants in these organisms has raised concerns regarding animal health, environmental contamination, and the potential transmission of resistant bacteria through the food chain (10). In addition, commensal Enterobacterales have been widely used as indicators of antimicrobial resistance pressure in animal production systems because of their capacity to accumulate and disseminate resistance determinants even in the absence of clinical disease (11).

Poultry production systems constitute important interfaces for the maintenance and circulation of antimicrobial-resistant Enterobacterales due to frequent interactions among animals, humans, and environmental matrices such as litter, water, and soil (3, 4). Turkeys (*Meleagris gallopavo*) represent a poultry species of major economic importance worldwide, with production systems ranging from large-scale industrial operations to smaller commercial settings. Turkey production is also economically relevant in Peru, where national production reached 41.6 thousand tons in 2024 and increased to 51.9 thousand tons in 2025, with an estimated per capita consumption of 1.45 kg in 2025 (12). Characteristics such as antimicrobial exposure, animal density, and environmental contamination may facilitate the persistence and dissemination of multidrug-resistant bacteria within these systems (13, 14). Despite this, antimicrobial resistance dynamics in turkey production environments remain insufficiently characterized, particularly regarding the co-occurrence of phenotypic resistance profiles across animal and environmental compartments.

Conventional surveillance approaches for antimicrobial resistance are primarily based on descriptive analyses of individual antimicrobials and may have limited ability to characterize multidimensional resistance patterns across bacterial isolates (15). Although these approaches are useful for surveillance, they may fail to identify phenotypic similarities and co-occurrence patterns among resistance traits. In this context, exploratory multivariate approaches may provide complementary information for understanding phenotypic resistance organization. Multiple correspondence analysis (MCA), a dimensionality reduction technique suitable for categorical data, allows visualization of associations among resistance variables and identification of groups of isolates sharing similar phenotypic profiles. When combined with clustering methods, MCA may facilitate exploratory characterization of resistance patterns beyond individual antimicrobial frequencies (16).

Although MCA has been applied in veterinary epidemiology to evaluate diagnostic and resistance profiles in selected pathogens (17), previous studies using multivariate analytical approaches have also demonstrated their utility for identifying distinct antimicrobial resistance phenotypes within bacterial populations (18). Nevertheless, the application of these approaches to characterize phenotypic antimicrobial resistance patterns among Enterobacterales recovered from poultry production environments remains limited. Therefore, this study aimed to characterize phenotypic antimicrobial resistance patterns and explore resistance clustering among Enterobacterales isolated from turkeys and their production environment in Lima, Peru.

## Methodology

### Study context and design

A descriptive cross-sectional study was conducted to characterize phenotypic antimicrobial resistance patterns among Enterobacterales isolated from turkeys (*Meleagris gallopavo domesticus*) and their production environment. The unit of analysis was the bacterial isolate. The study focused on lactose-fermenting Enterobacterales recovered from cloacal and environmental samples. Sampling was conducted during April 2026 in a semi-technified small-scale turkey production system located in Lima, Peru. The farm was selected by convenience based on accessibility and the owner's willingness to participate. It was located in a mixed agricultural–livestock area where pigs, cattle, and other poultry were also raised. The farm, which focused on meat production, housed 8,000 birds raised in confined conditions under routine commercial practices and used azithromycin as a growth promoter.

### Sampling strategy and sample collection

A convenience sampling strategy was applied during a single cross-sectional sampling visit conducted in April 2026. A total of 25 samples were collected, comprising 19 cloacal swabs obtained from 19 individual turkeys and six environmental litter samples collected from poultry houses across the production system. Up to two Enterobacterales isolates were recovered from each sample, yielding 50 isolates overall: 38 from cloacal samples and 12 from environmental samples. Cloacal samples were obtained by trained personnel using standardized handling procedures to minimize animal stress and ensure sample consistency. Environmental litter samples were aseptically collected from multiple points within each poultry house to include different areas of environmental exposure. All samples were placed in Cary-Blair transport medium and transported to the laboratory for processing within 24 h of collection.

### Bacteria isolation and identification

Cloacal and litter samples were directly inoculated onto MacConkey agar and incubated at 37 °C for 18–24 h. Lactose-fermenting colonies were selected as presumptive Enterobacterales. Up to two colonies per sample were selected for further identification when differences in colony morphology were observed, in order to capture phenotypically distinct presumptive Enterobacterales within the same sample. Presumptive isolates were screened based on colony morphology, lactose fermentation, and oxidase negativity. Bacterial identification was performed using conventional biochemical methods, including triple sugar iron agar (TSI), lysine iron agar (LIA), citrate utilization, and sulfide–indole–motility (SIM) tests. Isolates confirmed as Enterobacterales were included in the antimicrobial susceptibility analysis.

### Antimicrobial susceptibility testing

Antimicrobial susceptibility testing was performed using the Kirby–Bauer disk diffusion method on Mueller–Hinton agar, following standardized procedures. The antimicrobial panel included ceftazidime (30 μg), aztreonam (30 μg), gentamicin (10 μg), cefepime (30 μg), ceftriaxone (30 μg), amoxicillin–clavulanic acid (30 μg), cefuroxime (30 μg), ciprofloxacin (5 μg), chloramphenicol (30 μg), tetracycline (30 μg), nalidixic acid (30 μg), cefoxitin (30 μg), amikacin (30 μg), cefotaxime (30 μg), ertapenem (10 μg), ampicillin (10 μg), cephalothin (30 μg), imipenem (10 μg), meropenem (10 μg), azithromycin (15 μg), piperacillin–tazobactam (110 μg), ampicillin–sulbactam (20 μg), sulfamethoxazole–trimethoprim (25 μg), amoxicillin (25 μg), tobramycin (10 μg), kanamycin (30 μg), and neomycin (30 μg). *Escherichia coli* ATCC 25922 was used as the quality control strain for antimicrobial susceptibility testing. The panel was selected to capture a broad spectrum of resistance phenotypes across clinically and epidemiologically relevant antimicrobial classes. Interpretation of inhibition zone diameters was performed according to Clinical and Laboratory Standards Institute (CLSI) criteria (19). EUCAST clinical breakpoints were applied exclusively to azithromycin, as CLSI does not routinely provide interpretative criteria for this antimicrobial in Enterobacterales (20). Intermediate results were grouped with susceptible results for analytical purposes (19). Phenotypic detection of extended-spectrum β-lactamase (ESBL) production was performed using a synergy-based disk diffusion method on Mueller–Hinton agar. ESBL confirmation was conducted using the double-disk synergy test according to current EUCAST recommendations (20), with amoxicillin–clavulanic acid placed at the center and cefotaxime, ceftazidime, cefepime, and aztreonam positioned at a distance of 20 mm (center-to-center).

### Data analysis

The variables included in the analysis were phenotypic antimicrobial resistance profile, multidrug resistance (MDR), extended-spectrum β-lactamase (ESBL) production, bacterial species, and sampling source. Antimicrobial resistance was evaluated at both the individual antimicrobial level and aggregated by antimicrobial class. ESBL production was defined as a binary variable (positive/negative). Multidrug resistance (MDR) was defined as resistance to three or more antimicrobial classes according to the criteria proposed by Magiorakos et al. (21). Sampling source was categorized according to specimen origin (cloacal swab or environmental sample).

Descriptive analyses were conducted for all variables and expressed as absolute and relative frequencies. For all tested antimicrobials, reported values correspond exclusively to resistant isolates. In addition, the number of resistant antimicrobials and the number of resistant antimicrobial classes per isolate were calculated to characterize the overall resistance burden. Associations between bacterial species and sampling source with MDR status and resistance to individual antimicrobials were evaluated using Fisher's exact test due to small sample sizes and sparse data. Differences in the number of resistant antimicrobials and resistant antimicrobial classes across bacterial species and sampling sources were assessed using non-parametric tests (Kruskal–Wallis and Mann–Whitney *U* tests) due to the non-normal distribution of count variables. Phenotypic resistance combinations among quinolone- and aminoglycoside-resistant isolates were interpreted according to previously described phenotype-based resistance patterns reported for Gram-negative bacteria. These phenotypic profiles were used as exploratory indicators of putative resistance mechanisms, including target-site mutations, plasmid-mediated quinolone resistance determinants, and aminoglycoside-modifying enzymes (22). To explore phenotypic resistance combinations across isolates, a heatmap was generated to visualize antimicrobial resistance profiles and identify recurrent multidrug resistance patterns.

Multiple correspondence analysis (MCA) was applied to explore phenotypic resistance patterns among isolates. Each bacterial isolate represented one analytical unit, while antimicrobial resistance classes were included as active categorical variables. Resistance variables were dichotomized as resistant vs. non-resistant, with isolates categorized as susceptible or intermediate grouped as non-resistant for the MCA. MCA was selected because of its suitability for categorical data and its ability to identify patterns of co-occurrence among resistance variables. The number of retained dimensions was evaluated considering the eigenvalues, percentage of explained inertia, cumulative inertia, and interpretability of the dimensions. Based on these criteria, the first two dimensions were retained for subsequent clustering analyses, accounting for 45.3% of the total inertia (Dimension 1: 25.6%; Dimension 2: 19.7%). Eigenvalues, percentage of inertia, and cumulative inertia for all MCA dimensions are presented in Supplementary Table 2.

Individual coordinates derived from the first two MCA dimensions were used as input for k-means clustering. Clustering was performed using the Hartigan–Wong algorithm with 50 random initializations to reduce the likelihood of convergence to local minima. The optimal number of clusters was initially evaluated using average silhouette width, while the final solution was selected by jointly considering cluster separation, cluster size, stability assessed through bootstrap resampling (100 iterations) and quantified using the Jaccard similarity coefficient, and biological and epidemiological interpretability. Higher Jaccard similarity values indicated greater cluster stability.

All statistical analyses were conducted using Stata/SE version 18.5 (StataCorp, College Station, TX, USA), while multivariate analyses and graphical visualization were performed in R version 4.5.2 (R Foundation for Statistical Computing, Vienna, Austria). MCA was performed using the FactoMineR package; factoextra and ggplot2 were used for graphical visualization; fpc was used to assess cluster stability through bootstrap resampling and Jaccard similarity; and dplyr was used for data preprocessing and transformation.

## Results

### Antimicrobial susceptibility and resistance profiles

A total of 50 Enterobacterales isolates were recovered from cloacal swabs of turkeys (*n* = 38, 76.0%) and environmental samples (*n* = 12, 24.0%). The isolates were predominantly *Escherichia coli* (*n* = 39, 78.0%), followed by *Klebsiella* spp. (*n* = 7, 14.0%) and *Citrobacter* spp. (*n* = 4, 8.0%). All isolates met the criteria for multidrug resistance (MDR). Resistance to aminoglycosides and tetracyclines was highly prevalent, with universal resistance to kanamycin (*n* = 50, 100.0%), as well as high resistance to tetracycline (*n* = 48, 96.0%) and neomycin (*n* = 42, 84.0%). Among *E. coli* isolates, high resistance frequencies were observed for tetracycline (100.0%), neomycin (89.7%), and cefuroxime (71.8%). *Klebsiella* spp. isolates showed high resistance to cefuroxime (100.0%), tetracycline (85.7%), and amoxicillin–clavulanic acid (85.7%). All *Citrobacter* spp. isolates were resistant to amoxicillin–clavulanic acid and cephalothin. Carbapenem resistance was observed in isolates of both *E. coli* and *Citrobacter* spp. None of the isolates exhibited a phenotypic ESBL-positive result (Table 1).

**Table 1:** Phenotypic antimicrobial resistance profiles and source distribution of enterobacterial isolates.

Results	Total (*n* = 50)	Bacterial species			*p*-value^*^
		*E. coli* (*n* = 39)	*Klebsiella* spp. (*n* = 7)	*Citrobacter* spp. (*n* = 4)	
Sample source					
Cloacal swab	38 (76.00)	31 (79.49)	5 (71.43)	2 (50.00)	0.356
Environmental samples	12 (24.00)	8 (20.51)	2 (28.57)	2 (50.00)	
*β*-lactam					
Ampicillin	28 (56.00)	20 (51.28)	5 (71.43)	3 (75.00)	0.829
Amoxicillin	28 (56.00)	20 (51.28)	5 (71.43)	3 (75.00)	0.829
*β*-lactam/inhibitor					
Amoxicillin/Clavulanic Acid	36 (72.00)	26 (66.67)	6 (85.71)	4 (100.00)	0.843
Ampicillin/Sulbactam	4 (8.00)	1 (2.56)	0 (0.00)	3 (75.00)	0.006
Piperacillin/Tazobactam	2 (4.00)	0 (0.00)	0 (0.00)	2 (50.00)	0.005
Cephalosporins					
Cephalothin	17 (34.00)	9 (23.08)	4 (57.14)	4 (100.00)	0.019
Cefuroxime	38 (76.00)	28 (71.79)	7 (100.00)	3 (75.00)	0.231
Cefoxitin	3 (6.00)	2 (5.13)	1 (14.29)	0 (0.00)	0.643
Cefotaxime	8 (16.00)	4 (10.26)	2 (28.57)	2 (50.00)	0.039
Ceftazidime	7 (14.00)	3 (7.69)	1 (14.29)	3 (75.00)	0.002
Ceftriaxone	5 (10.00)	3 (7.69)	2 (28.57)	0 (0.00)	0.180
Cefepime	4 (8.00)	3 (7.69)	1 (14.29)	0 (0.00)	0.444
Monobactams					
Aztreonam	3 (6.00)	1 (2.56)	0 (0.00)	2 (50.00)	0.063
Carbapenems					
Imipenem	3 (6.00)	0 (0.00)	0 (0.00)	3 (75.00)	< 0.001
Meropenem	2 (4.00)	2 (5.13)	0 (0.00)	0 (0.00)	0.005
Ertapenem	0 (0.00)	0 (0.00)	0 (0.00)	0 (0.00)	N.A.
Aminoglycosides					
Gentamycin	6 (12.00)	4 (10.26)	2 (28.57)	0 (0.00)	0.423
Amikacin	0 (0.00)	0 (0.00)	0 (0.00)	0 (0.00)	N.A.
Tobramycin	5 (10.00)	4 (10.26)	1 (14.29)	0 (0.00)	0.005
Kanamycin	50 (100.0)	39 (100.0)	7 (100.0)	4 (100.0)	N.A.
Neomycin	42 (84.00)	35 (89.75)	5 (71.43)	2 (50.00)	0.042
Quinolones					
Nalidixic Acid	13 (26.00)	7 (17.95)	3 (42.86)	3 (75.00)	0.122
Ciprofloxacin	13 (26.00)	8 (20.51)	2 (28.75)	3 (75.00)	0.094
Tetracyclines					
Tetracycline	48 (96.00)	39 (100.0)	6 (85.71)	3 (75.00)	0.028
Macrolides					
Azithromycin	17 (34.00)	11 (28.21)	3 (42.86)	3 (75.00)	0.145
Amphenicols					
Chloramphenicol	16 (32.00)	14 (38.46)	1 (14.29)	0 (0.00)	0.001
Sulfonamides					
Sulfamethoxazole/Trimethoprim	3 (6.00)	1 (2.56)	0 (0.00)	2 (50.00)	0.042

Values correspond to the frequency [*n* (%)] of resistant isolates across all tested antimicrobials.

^*^P-values correspond to comparisons of resistance frequencies across bacterial species using Fisher's exact test.

N.A., not applicable because no variability in resistance status was observed.

Resistance burden varied across bacterial species. Overall, 24.0% (*n* = 12) of the isolates were resistant to 10 or more antimicrobials, including six *E. coli*, three *Klebsiella* spp., and three *Citrobacter* spp. isolates. The isolate with the broadest resistance profile was an *E. coli* recovered from a cloacal swab, which exhibited resistance to 18 antimicrobials, including aminopenicillins, β-lactam/β-lactamase inhibitor combinations, and multiple cephalosporins. Similarly, a *Citrobacter* spp. isolate recovered from an environmental sample showed resistance to 17 antimicrobials, including imipenem, aminopenicillins, β-lactam/β-lactamase inhibitor combinations, and piperacillin–tazobactam. The most frequently observed resistance profile comprised kanamycin, tetracycline, neomycin, cefuroxime, and amoxicillin–clavulanic acid. *Citrobacter* spp. consistently showed higher resistance burdens than *E. coli* and *Klebsiella* spp., although differences did not reach statistical significance (Figure 1).

**Figure 1:**
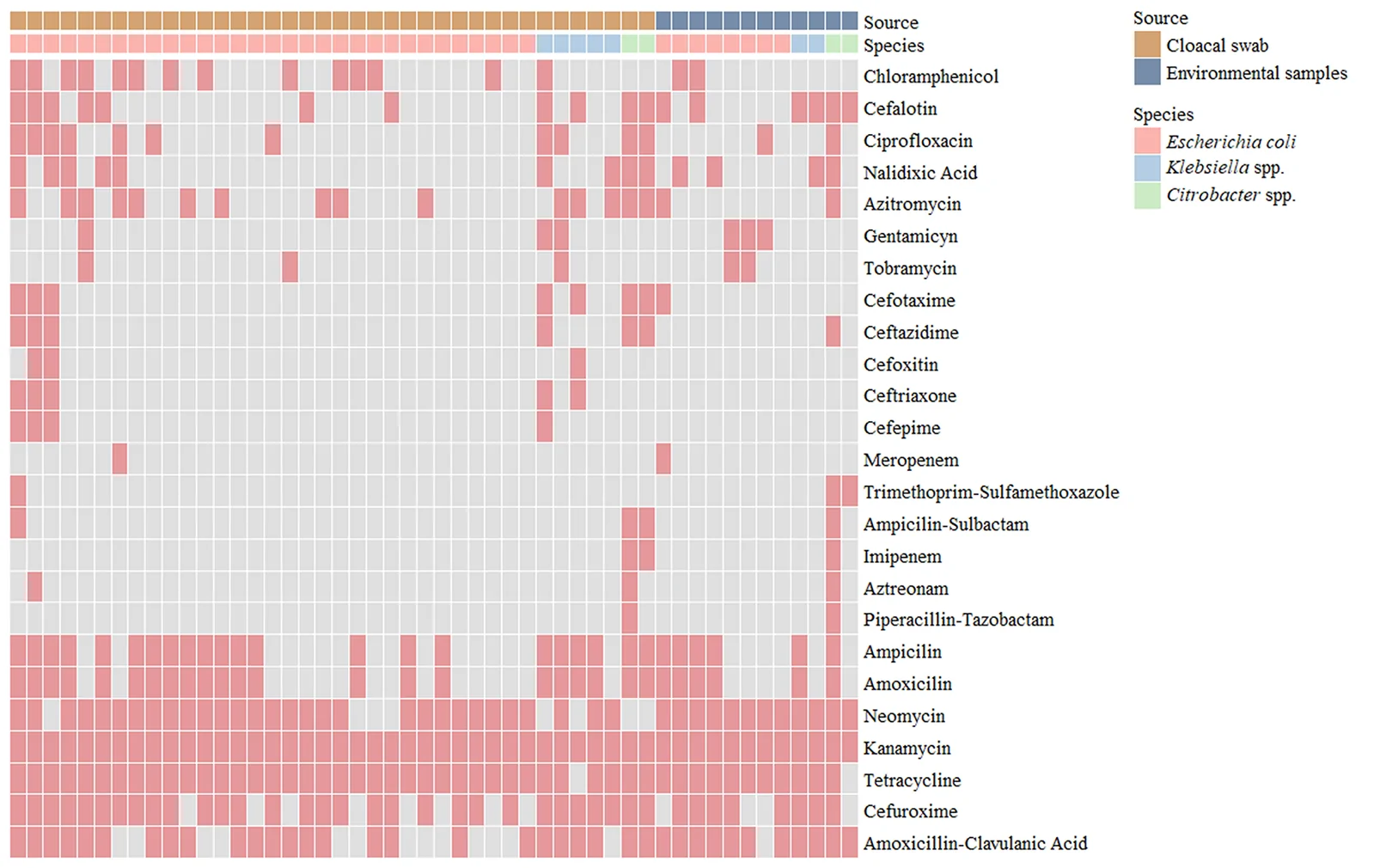
Visualization of individual antimicrobial resistance profiles across 26 agents for enterobacterial isolates.

Several antimicrobial resistance patterns were identified in relation to both phenotypic and genotypic putative resistance mechanisms. The most frequent combination within the quinolone class corresponded to resistance to nalidixic acid (NA) and ciprofloxacin (CIP), observed in 16.0% (*n* = 8) of the isolates, including four *E. coli*, one *Klebsiella spp*., and three *Citrobacter spp*. isolates. In addition, complex phenotypic resistance patterns were identified within the aminoglycoside class, with the most prevalent being the combination of gentamicin (GN), amikacin (AK), tobramycin (TO), kanamycin (K), and neomycin (N), present in 36 (72.0%) isolates. *E. coli* was the primary carrier of this phenotype, accounting for 76.92% (*n* = 30). These resistance profiles may be indicative of distinct quinolone and aminoglycoside resistance mechanisms; however, molecular confirmation was not performed in the present study (Table 2).

**Table 2:** Phenotypic resistance combinations associated with putative resistance mechanisms.

Results		Total (*n* = 50)	Bacteria species	
Phenotype-based inferred mechanism	Phenotype combination		*Escherichia coli* (*n* = 39)	*Klebsiella spp*. (*n* = 7)	*Citrobacter spp*. (*n* = 4)
Quinolones					
gyrA	NA(R) +CIP(S)	2 (4.00)	2 (5.13)	0 (0.00)	0 (0.00)
gyrA/gyrA + parC	NA(R) + CIP(I)	3 (6.00)	1 (2.56)	2 (28.57)	0 (0.00)
qnr	NA(R) +CIP(R)	8 (16.00)	4 (10.26)	1 (14.29)	3 (75.00)
Aminoglycosides					
AAC(3)IV	GN(R) +AK(S) + TO(R) + K(R) + N(R)	4 (8.00)	3 (7.69)	1 (14.29)	0 (0.00)
APH(3')	GN(S) +AK(S) + TO(S) + K(R) + N(R)	36 (72.00)	30 (76.92)	4 (57.14)	2 (50.00)

R, resistant; I, intermediate; S, susceptible; NA, nalidixic acid; CIP, ciprofloxacin; GN, Gentamycin; AK, amikacin; TO, tobramycin; K, kanamycin; N, neomycin.

The distribution of antimicrobial resistance was evaluated according to bacterial species and sampling source. Isolates of *Citrobacter* spp. showed higher resistance rates to antibiotics and antibiotic classes compared to those of *Klebsiella* spp. and *E. coli*. However, these differences were not statistically significant according to the Kruskal–Wallis test (*p* = 0.051 and *p* = 0.181, respectively). Regarding the sampling source, no differences were observed in the distribution of the number of antibiotics or classes of antibiotics between cloacal and environmental samples, which was consistent with the results of the Mann–Whitney *U* test (*p* = 0.671 and *p* = 0.427, respectively; Figure 2).

**Figure 2:**
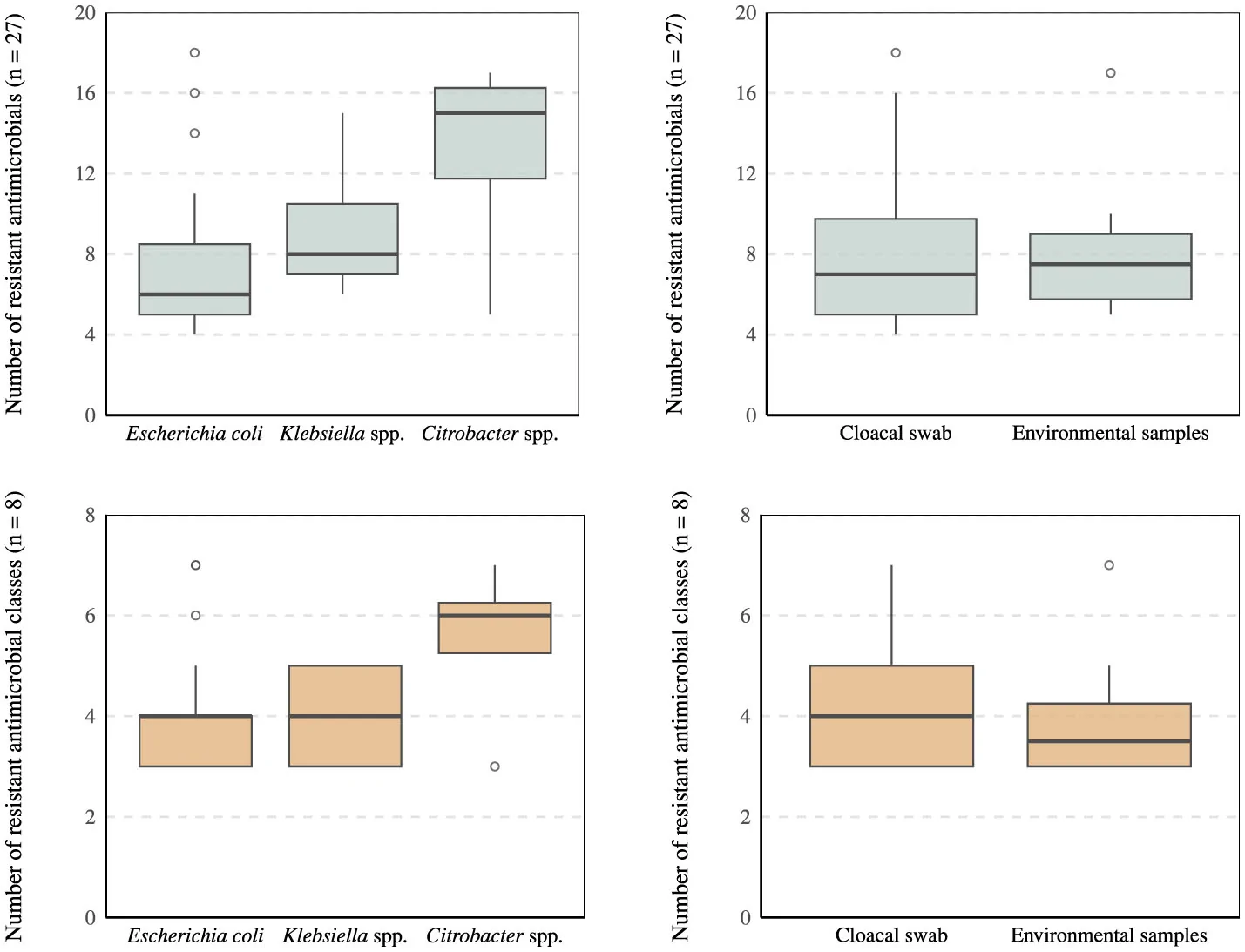
Distribution of antimicrobial resistance counts by bacterial species and sampling source.

### Dimensional structure and clustering of phenotypic resistance profiles

Multiple correspondence analysis (MCA) identified seven dimensions. The first two dimensions accounted for 45.3% of the total inertia (Dimension 1: 25.6%; Dimension 2: 19.7%). Although additional dimensions increased the cumulative inertia, exploratory clustering analyses indicated that they did not improve the stability or epidemiological interpretability of the resulting resistance profiles. Therefore, the first two dimensions were retained for subsequent clustering analyses and graphical representation of the phenotypic resistance structure.

Resistance to carbapenems (CA), quinolones (Q), and macrolides (MA) was projected toward the positive region of Dimension 1, whereas resistance to tetracyclines (TE) and sulfonamides (SF) contributed predominantly to Dimension 2 (Figure 3). The spatial distribution of antimicrobial classes suggested distinct patterns of co-occurrence among resistance phenotypes. Based on the individual coordinates derived from the MCA, three clusters were identified. Cluster 1 was concentrated in the negative region of Dimension 1 and exhibited limited dispersion. Cluster 2 occupied an intermediate position, with a broader distribution across both dimensions. Cluster 3 showed the greatest dispersion and was located predominantly in the positive region of Dimension 1, indicating greater variability in phenotypic resistance profiles (Figure 3).

**Figure 3:**
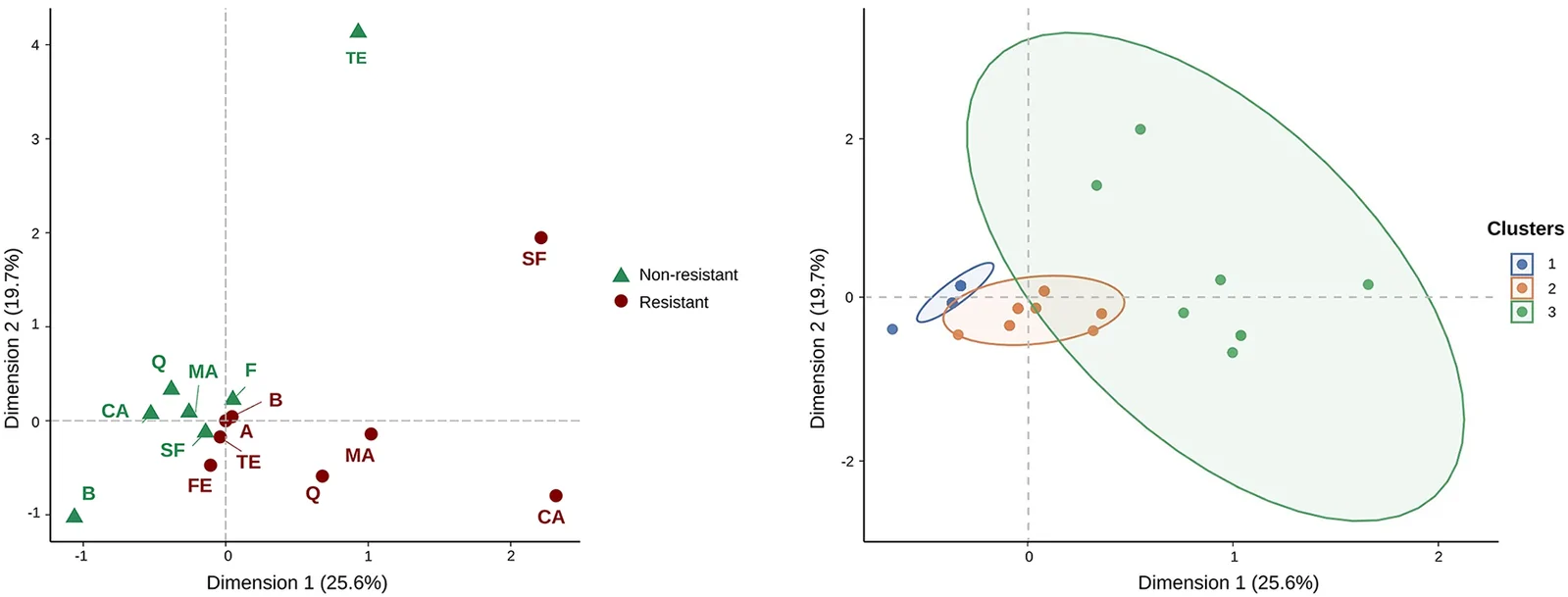
Dimensional structure and clustering of resistance phenotypic profiles by multiple correspondence analysis in enterobacterial isolates. B, beta-lactams; CA, carbapenems; Q, quinolones; A, aminoglycosides; SF, sulfonamides; TE, tetracyclines; FE, phenicols; MA, macrolides.

### Cluster validation and stability analysis

Cluster solutions were evaluated using silhouette width and bootstrap-based stability analyses. Although solutions with a larger number of clusters yielded slightly higher silhouette values, they resulted in fragmented groups with limited epidemiological interpretability. The three-cluster solution achieved an average silhouette width of 0.68, representing an appropriate balance between statistical performance and biological relevance. Bootstrap resampling (100 iterations) indicated that the three-cluster solution was more stable than the four-cluster alternative, achieving a higher average Jaccard similarity coefficient (0.72 vs. 0.66). In contrast, the four-cluster solution contained a highly unstable cluster (Jaccard = 0.32). Considering cluster cohesion, relative stability, and epidemiological interpretability, the three-cluster solution was selected for subsequent analyses. The final solution comprised 22, 20, and 8 isolates in Clusters 1, 2, and 3, respectively.

### Phenotypic patterns of antimicrobial resistance and cluster identification

Distinct resistance patterns were observed across clusters (Table 3). Cluster 1 was characterized by high frequencies of resistance to tetracyclines (100.0%), aminoglycosides (100.0%), and beta-lactams (95.5%), together with moderate resistance to phenicols (31.8%). No resistance to macrolides, sulfonamides, quinolones, or carbapenems was detected in this cluster. Cluster 2 exhibited a similar resistance profile, with high resistance to tetracyclines (100.0%), aminoglycosides (100.0%), and beta-lactams (95.0%), but also included moderate frequencies of resistance to quinolones (65.0%), macrolides (50.0%), and phenicols (35.0%). Cluster 3 displayed the broadest resistance spectrum, with universal resistance to beta-lactams (100.0%) and aminoglycosides (100.0%), accompanied by resistance to macrolides (87.5%), tetracyclines (75.0%), carbapenems (62.5%), quinolones (62.5%), sulfonamides (37.5%), and phenicols (25.0%). Notably, this was the only cluster in which carbapenem resistance was identified. The phenotypic subprofiles identified within each cluster are presented in Supplementary Table 1. Cluster 1 was dominated by a single resistance profile (*n* = 15; 68.2%), indicating a high degree of internal homogeneity. In contrast, Clusters 2 and 3 each comprised seven distinct phenotypic profiles. While Cluster 2 exhibited a moderate distribution of subprofiles, Cluster 3 showed greater dispersion, with each subprofile represented by no more than two isolates, reflecting increased phenotypic heterogeneity within this group.

**Table 3:** Phenotypic characterization of clusters using multiple correspondence analysis in enterobacterial isolates.

Phenotypes	B	CA	Q	A	SF	TE	FE	MA
Cluster 1 (*n* = 22)	95.5	0.0	0.0	100.0	0.0	100.0	31.8	0.0
Cluster 2 (*n* = 20)	95.0	0.0	65.0	100.0	0.0	100.0	35.0	50.0
Cluster 3 (*n* = 8)	100.0	62.5	62.5	100.0	37.5	75.0	25.0	87.5

B, beta-lactams; CA, carbapenems; Q, quinolones; A, aminoglycosides; SF, sulfonamides; TE, tetracyclines; FE, phenicols; MA, macrolides.

## Discussion

This study identified widespread antimicrobial resistance among Enterobacterales recovered from turkeys and their production environment in a small-scale poultry system. All isolates met the criteria for multidrug resistance, and high frequencies of resistance were observed to aminoglycosides and tetracyclines. In addition, a subset of isolates exhibited phenotypic carbapenem resistance, including one *Citrobacter* spp. isolate with resistance to a broad range of antimicrobial classes. The multivariate analysis further revealed that resistance phenotypes were not randomly distributed but rather organized into distinct clusters, suggesting the coexistence of different phenotypic resistance patterns within the production system.

This study took place in a small-scale poultry production system, a setting that differs from industrial operations in biosecurity, animal density, and regulatory oversight. These systems are increasingly seen as an overlooked driver of antimicrobial resistance emergence and spillover into the community (23). Resistance to aminoglycosides and tetracyclines was the most consistent finding across bacterial species. High levels of resistance to tetracycline, kanamycin, and neomycin were particularly evident among *E. coli* isolates, partially coinciding with findings reported in clinical turkey isolates from Hungary, where elevated levels of neomycin resistance were also observed (24). Likewise, multidrug-resistant *E. coli* have been reported in healthy turkeys from Bangladesh, although that study documented substantially higher levels of meropenem resistance than those identified here (25). Differences between studies may reflect variation in antimicrobial use practices, production systems, local selective pressures, and regional regulatory frameworks. Nevertheless, the recurrent detection of resistance to older and commonly used antimicrobial classes across geographically distant poultry systems suggests persistent selective pressure acting on commensal Enterobacterales populations.

Among *Klebsiella* spp., resistance to cefuroxime, tetracycline, and amoxicillin–clavulanic acid was particularly frequent. Similar resistance profiles have been reported in poultry-associated *Klebsiella* isolates from Pakistan and Bangladesh, including complete resistance to cefuroxime and high levels of resistance to tetracyclines (26, 27). Resistance to amoxicillin–clavulanic acid has also been documented in poultry production chains, although reported frequencies vary considerably according to geographic region and production practices (28). Together, these findings support the growing recognition of poultry systems as reservoirs of *Klebsiella* spp. carrying clinically relevant resistance phenotypes and highlighting the importance of including this genus in antimicrobial resistance surveillance programs. *Klebsiella* spp. are increasingly recognized as relevant reservoirs of antimicrobial resistance at the animal–human interface. Multidrug-resistant strains have been reported in poultry and in humans with occupational exposure to poultry production environments (29). In addition, *Klebsiella pneumoniae* has been reported more frequently in turkeys than in broiler chickens, suggesting that turkey production may represent a relevant reservoir requiring further investigation (30). These findings support the inclusion of *Klebsiella* spp. in antimicrobial resistance surveillance across poultry production chains, particularly within a One Health framework addressing potential connections between animal and human resistance reservoirs.

The findings involving *Citrobacter* spp. deserve particular attention. One isolate recovered from the production environment exhibited resistance to 17 antimicrobials, including imipenem. Although carbapenem-resistant *Citrobacter* spp. have been reported in clinical settings (31) and environmental sources (32), information regarding their occurrence in turkey production systems remains scarce. Previous studies have documented carbapenem-resistant Enterobacterales in animals and poultry-associated environments (33, 34); however, carbapenems are not routinely used in veterinary medicine (35). The detection of phenotypic carbapenem resistance in this context is noteworthy given the limited use of carbapenems in veterinary medicine (36). Although the present study did not investigate the origin or molecular mechanisms underlying this resistance, several potential pathways have been described at the animal–human–environment interface. Environmental contamination, particularly through wastewater and other anthropogenic sources, may contribute to the introduction and circulation of carbapenem-resistant Enterobacterales and their resistance determinants in agricultural environments (37). Contaminated water, feed, wild birds, and other environmental vectors have also been implicated in the dissemination of antimicrobial-resistant bacteria within and between poultry production systems (38). However, none of these pathways can be established from the present findings and should therefore be considered plausible hypotheses rather than demonstrated sources of the resistance observed in this study. Molecular characterization of the isolates, together with environmental source-tracking approaches, will be necessary to determine the mechanisms and potential sources underlying the observed carbapenem resistance.

From a One Health perspective, the identification of multidrug-resistant Enterobacterales in both cloacal and environmental samples is epidemiologically relevant. Enterobacterales resistant to critically important antimicrobials may circulate across animal, human, and environmental compartments, contributing to the maintenance and dissemination of resistance determinants. This concern is particularly relevant for carbapenems, which remain among the most important therapeutic options for severe infections caused by multidrug-resistant Gram-negative bacteria and are considered critically important for human medicine by the World Health Organization (39). Although carbapenems are not authorized for veterinary use under current international recommendations (40), resistance to these agents has increasingly been reported in non-clinical environments, suggesting that transmission pathways extend beyond direct antimicrobial exposure (41). Consequently, the presence of phenotypic carbapenem-resistant Enterobacterales in food-producing animals reinforces the need for integrated surveillance strategies encompassing animal, environmental, and human reservoirs. Similar resistance patterns have been documented in poultry production systems elsewhere in Latin America, including ESBL/AmpC-producing Escherichia coli in Brazil and extended-spectrum cephalosporin-resistant Salmonella enterica in the Colombian poultry chain (42, 43). In Peru, antimicrobial-resistant E. coli has been reported in poultry, together with mcr-1-harboring E. coli from chicken and pig farms in Lima (44, 45). This evidence is particularly relevant in the context of Peru's National Multisectoral Action Plan on Antimicrobial Resistance, which incorporates a One Health approach and promotes coordinated surveillance across human, animal, and environmental sectors (46). However, evidence from turkey production remains limited compared with that available for broiler chickens. The present study contributes data from this comparatively understudied poultry production setting and supports the inclusion of diverse poultry systems in integrated antimicrobial resistance surveillance.

An important contribution of this study was the application of multiple correspondence analysis to characterize the phenotypic organization of antimicrobial resistance. Conventional surveillance approaches frequently focus on the prevalence of resistance to individual antimicrobials; however, they may overlook relationships among resistance traits. In the present study, MCA revealed a structured distribution of resistance phenotypes and identified clusters that differed in their resistance spectrum. Notably, one cluster concentrated isolates exhibiting resistance to carbapenems, quinolones, and macrolides, whereas other clusters were characterized primarily by resistance to beta-lactams, aminoglycosides, and tetracyclines. These findings suggest that antimicrobial resistance within the production system is composed of multiple phenotypic profiles rather than a single homogeneous multidrug-resistant population. The identification of these resistance structures highlights the value of exploratory multivariate approaches for understanding antimicrobial resistance patterns in animal production systems and may contribute to the development of more targeted surveillance strategies (47, 48).

This study has several limitations. First, the non-probabilistic sampling strategy, the relatively small number of isolates, and the inclusion of a single small-scale poultry production system limit the representativeness of the findings and their generalizability to other production settings or geographic regions. Subgroup sizes were particularly small for *Citrobacter* spp. (*n* = 4) and environmental isolates (*n* = 12), limiting robust statistical inference for these categories. Second, the cross-sectional design did not allow assessment of temporal changes in antimicrobial resistance. Detailed information on antimicrobial use was unavailable beyond the reported use of azithromycin as a growth promoter, which prevented assessment of potential associations between antimicrobial exposure and the observed resistance patterns. Bacterial identification relied on conventional phenotypic and biochemical methods, limiting species-level discrimination for some Enterobacterales; consequently, Klebsiella and Citrobacter isolates were conservatively reported at the genus level. Third, confirmatory phenotypic or molecular assays for carbapenemase production, such as mCIM/eCIM, CarbaNP, or molecular detection of carbapenemase genes, were not performed. Therefore, the carbapenem resistance observed in this study should be interpreted strictly as a phenotypic finding based on disk diffusion and not as evidence of carbapenemase production or any specific resistance mechanism. More broadly, the phenotypic approach used in this study does not allow intrinsic and acquired resistance mechanisms to be distinguished. Further studies incorporating confirmatory phenotypic and molecular or genomic approaches are needed to characterize the mechanisms underlying the observed carbapenem resistance. Finally, MCA is an exploratory multivariate technique and does not establish causal relationships or identify the biological mechanisms underlying the observed resistance profiles. Isolates assigned to the same cluster should therefore not be assumed to share antimicrobial resistance genes, plasmids, or other mobile genetic elements; rather, the clusters represent phenotypic resistance patterns that tend to co-occur. Because the clustering structure was derived from isolates recovered from a single production system, its reproducibility should be evaluated in larger studies encompassing different poultry production systems and geographic regions.

## Conclusions

Enterobacterales isolated from turkeys and their production environment exhibited widespread multidrug resistance and a heterogeneous distribution of phenotypic resistance profiles across animal and environmental compartments. To our knowledge, this is the first study to apply multiple correspondence analysis to characterize the multivariate structure of phenotypic antimicrobial resistance in Enterobacterales from turkey production systems in Peru. This approach identified distinct resistance clusters that might not have been apparent through conventional analyses based solely on individual antimicrobials. The detection of phenotypic carbapenem resistance among a subset of isolates highlights the need to strengthen antimicrobial resistance surveillance and antimicrobial stewardship in small-scale poultry production settings, together with measures aimed at limiting the circulation of resistant bacteria between animals and their production environment. Given the limited sample size and evaluation of a single production system, these findings should be interpreted as preliminary and require validation in larger and more diverse poultry populations. Nevertheless, they support the potential value of commensal Enterobacterales as indicators of antimicrobial resistance patterns at the animal–environment interface and reinforce the relevance of integrated One Health surveillance. Larger and longitudinal studies incorporating molecular characterization are needed to validate the observed resistance patterns and characterize the mechanisms underlying the observed carbapenem resistance.

## Data Availability

All relevant data for this study are publicly available from the figshare repository (https://doi.org/10.6084/m9.figshare.32581647).

## Supplementary material

SUPPLEMENTARY TABLE 1 Patterns of antibiotic resistance using multiple correspondence analysis in enterobacterial isolates.

SUPPLEMENTARY TABLE 2 Inertia values and eigenvalues of the seven dimensions identified using multiple correspondence analysis in enterobacterial isolates.
